# Connection strength of the macaque connectome augments topological and functional network attributes

**DOI:** 10.1162/netn_a_00101

**Published:** 2019-09-01

**Authors:** Siemon C. de Lange, Dirk Jan Ardesch, Martijn P. van den Heuvel

**Affiliations:** Connectome Lab, Department of Complex Trait Genetics, Center for Neurogenomics and Cognitive Research, Vrije Universiteit Amsterdam, Amsterdam Neuroscience, Amsterdam, The Netherlands; Connectome Lab, Department of Complex Trait Genetics, Center for Neurogenomics and Cognitive Research, Vrije Universiteit Amsterdam, Amsterdam Neuroscience, Amsterdam, The Netherlands; Connectome Lab, Department of Complex Trait Genetics, Center for Neurogenomics and Cognitive Research, Vrije Universiteit Amsterdam, Amsterdam Neuroscience, Amsterdam, The Netherlands; Department of Clinical Genetics, Amsterdam UMC, Vrije Universiteit Amsterdam, Amsterdam Neuroscience, Amsterdam, The Netherlands

**Keywords:** Macaque, Connectome, Network, Projection strength, Functional synchronization

## Abstract

Mammalian brains constitute complex organized networks of neural projections. On top of their binary topological organization, the strength (or weight) of these neural projections can be highly variable across connections and is thus likely of additional importance to the overall topological and functional organization of the network. Here we investigated the specific distribution pattern of connection strength in the macaque connectome. We performed weighted and binary network analysis on the cortico-cortical connectivity of the macaque provided by the unique tract-tracing dataset of Markov and colleagues (2014) and observed in both analyses a small-world, modular and rich club organization. Moreover, connectivity strength showed a distribution augmenting the architecture identified in the binary network version by enhancing both local network clustering and the central infrastructure for global topological communication and integration. Functional consequences of this topological distribution were further examined using the Kuramoto model for simulating interactions between brain regions and showed that the connectivity strength distribution across connections enhances synchronization within modules and between rich club hubs. Together, our results suggest that neural pathway strength promotes topological properties in the macaque connectome for local processing and global network integration.

## Author Summary

High-resolution tract-tracing methods allow for the measurement of cortico-cortical anatomical pathways and quantification of their connectivity strength. The variable strength of neural pathways holds additional information on the complex organization of brain networks. We studied connectivity strength in the macaque by using the open-access connectome dataset of Markov and colleagues (2014), which contains high-resolution information on the number of axonal projections interconnecting brain regions. Our results show that connectivity strength adopts a nonrandom distribution and enhances key network features already present in the binary version of the network. Using the Kuramoto model to simulate functional dynamics, we further demonstrated that connectivity strength enhances local processing and global network integration. This study aids in the understanding of the macaque connectome by clarifying the role of connectivity strength in the network topology and functioning.

## INTRODUCTION

Brain function depends on efficient communication through a complex network of neural connections. A species’ macroscale connectome describes the total network of all anatomical communication pathways linking brain regions (Sporns et al., 2005). Tract-tracing methods enabled the mapping and reconstruction of comprehensive macroscale connectome maps of several mammalian species, including that of the cat (Scannell et al., 1999), the mouse (Oh et al., 2014; Zingg et al., 2014), the rat (Burns & Young, 2000; Swanson, 1992; Zakiewicz et al., 2011) and the macaque (Felleman & Van Essen, 1991; Markov et al., 2014; Modha & Singh, 2010; Stephan et al., 2001). Comparison of neural wiring topology across species has shown a tendency in neural networks toward modular organization providing functional specialization, and short communication paths, hubs, and rich club organization facilitating topological integration (van den Heuvel et al., 2016a). The connection strength of anatomical projections is noted to be strongly variable across pathways (Hilgetag & Grant, 2000; Markov et al., 2011), and graph analysis studies have shown that the connection strength distribution enhances functional specialization and global integration in the connectome of several mammals, including the rat (Bota et al., 2015; van den Heuvel et al., 2015), mouse (van den Heuvel & de Reus, 2014; Rubinov et al., 2015), and human brain (Grayson et al., 2014; Hagmann et al., 2008, 2010; van den Heuvel et al., 2012; van den Heuvel & Sporns, 2011).

High-resolution tract-tracing studies of the macaque have further employed systematic and standardized mapping of cortical white matter pathways (Markov et al., 2013b, 2014). These reconstructed macaque connectome maps include not only information on the presence and absence of macroscale anatomical projections but also detailed *quantitative* information on the *strength* of reconstructed anatomical projections. Studies investigating the dataset showed that the connection strength distribution adheres to an exponential decay in connection strength with connection length (Ercsey-Ravasz et al., 2013; Markov et al., 2013a) and that connections between brain regions sharing topological neighbors show high connectivity strength (Goulas et al., 2014). Network analyses further provided insight into the macaque connectome organization, elucidating a core that is important for network integration (Betzel et al., 2018; Ercsey-Ravasz et al., 2013; Markov et al., 2013a), a small-world organization (Bassett & Bullmore, 2017; Hilgetag & Goulas, 2015), and a modular organization (Song et al., 2014) providing complex functional dynamics (Honey & Sporns, 2008; Zamora-López et al., 2016). So far, network studies have been investigating the weighted and binary versions of the macaque connectome mostly separately, so the topological and functional consequences of the specific connectivity strength distribution in the macaque connectome remain unclear.

We examined the contribution of connectivity strength to the topological and functional organization of the macaque connectome. First, the binary macaque connectome was characterized with respect to key network organizational principles including small-world, modular, and rich club organization. Next, we tested whether the connection strength distribution enhances the identified organizational properties. Moreover, we related macaque connectome organization and connectivity strength distribution to functional synchrony between brain regions simulated by the Kuramoto model (Breakspear et al., 2010; Cabral et al., 2014; Rodrigues et al., 2016; Vlasov & Bifone, 2017; Vuksanović & Hövel, 2014, 2016). Our results indicate that connection strength across connections is distributed in such a way that it enhances the binary network organization, increasing local processing and global network integration.

## MATERIALS AND METHODS

### Macaque Connectome Data

**Figure 1.**
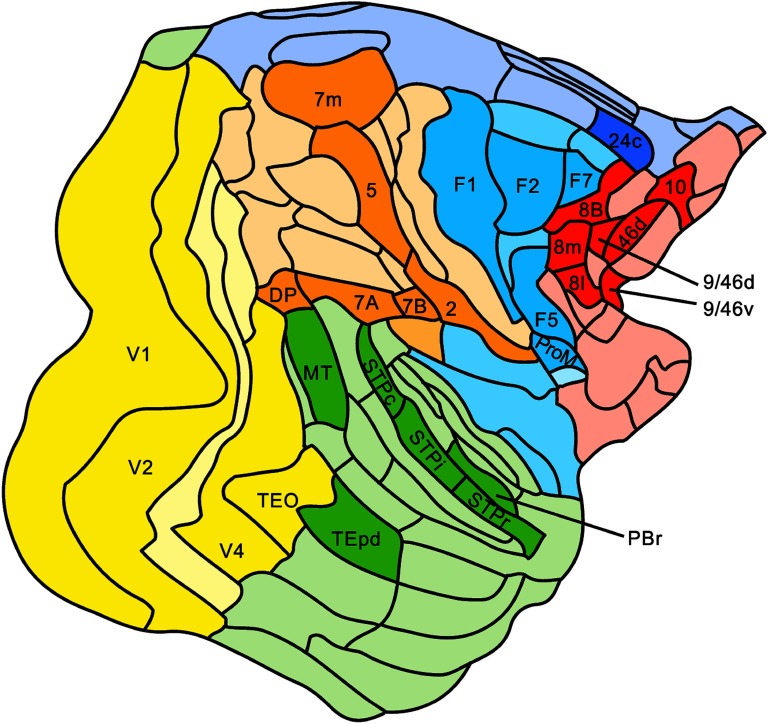
The 29 injection sites of the macaque dataset. Injection sites were distributed along all six cortical lobes, that is, 4 in occipital (yellow), 6 in temporal (green), 6 in parietal (orange), 5 in frontal (light blue), 7 in prefrontal (red), 1 in limbic (dark blue) cortical structures. The 29 injection regions are highlighted. The layout is as presented by Markov et al. (2014) and made available at core-nets.org by Markov and colleagues.

Anatomical connectivity of the monkey cortical connectome was obtained from the study by Markov and colleagues (2014). The macaque cortico-cortical structural connectivity dataset, as made freely available at http://core-nets.org (Markov et al., 2014), contains information collated from single-injection retrograde tract-tracing studies in 28 macaque specimens (27 *Macaca fascicularis*—left and right hemispheres intermixed—and 1 *Macaca mulatta*—right hemisphere) performed by Markov and coworkers. Retrograde viral tracer experiments were performed in 29 unique cortical regions, based on a reference atlas dividing the cortex into 91 cortical regions. Injection site regions (illustrated in Figure 1) were distributed over 4 areas in occipital, 6 in temporal, 6 in parietal, 5 in frontal, 7 in prefrontal and 1 area in limbic cortical structures. The dataset describes a dense cortical network consisting of 1,615 interareal pathways (Markov et al., 2014).

Following Markov et al. (2014), we examined the subnetwork of 29 injection sites (out of a total of 91 defined cortical regions) for which the dataset contains complete information on all possible axonal projections (Markov et al., 2014). The associated mathematical graph included 29 nodes, representing brain regions, and 536 directed connections, referring to axonal projections, forming a dense graph with 66% of the 812 possible connections present. Data was presented in the adjacency matrix *A*, with entry *A*_*ij*_ = 1 in case an axonal projection was reported between regions *i* and *j*, and *A*_*ij*_ = 0 if no projections were reported.

Besides information on presence or absence of pathways between brain regions, Markov et al. report on the number of labeled neurons (*p*_*ij*_) involved in each reported pathway. The number of labeled neurons formed a log-normal distribution, therefore the biologically more representative log-transformed number of labeled neurons, *w*_*ij*_ = log(*p*_*ij*_ + 1) was used as connectivity strength in the weighted network (Hagmann et al., 2008; Honey et al., 2009).

To compensate for fluctuations in effective tracer uptakes, Markov et al. also report the more relative measure of the extrinsic fraction of labeled neurons (FLNe), computed as the ratio between the number of labeled neurons and the total number of reported cortical neurons extrinsic to the injected area (Markov et al., 2014). Results from the FLNe-weighted network are presented in the Supporting Information.

### Network Measures

Network organizational characteristics were calculated for both binary and weighted networks, including the following metrics (Rubinov & Sporns, 2010).

*Clustering coefficient*. In the binary network, node-wise clustering coefficient *C*^*b*^ was computed, expressing the extent to which neighbors of a node are mutually connected (Watts & Strogatz, 1998). In weighted networks, the clustering coefficient *C*^*w*^ was examined, reflecting the strength of all closed triangles a node forms with its neighbors (Fagiolo, 2007). Both binary and weighted clustering coefficient were compared with the clustering coefficients $C_{random}^{b}$ and $C_{random}^{w}$ of randomized networks (10,000 randomizations examined), formed by randomly switching connections between regions, preserving the number of in- and outgoing connections (in- and out-degree), and total strength of the incoming connections (in-strength) of each node (Rubinov & Sporns, 2010). The ratios between the clustering coefficients of the original network and the clustering coefficient of randomized networks were denoted by the normalized clusterings $C_{norm}^{b}$ and $C_{norm}^{w}$. To assess the effect of the connectivity strength distribution on clustering, the weighted clustering coefficient was compared with the clustering coefficient $C_{shuffled}^{w}$ of 10,000 weights-shuffled network versions in which the connection strengths, as seen in the original network, were randomly redistributed.

*Shortest Path length.* Network integration was assessed by examining the shortest path length between nodes. The binary shortest path length between nodes is defined as the minimal number of connections needed to traverse from one node to another (Watts & Strogatz, 1998). The average binary shortest path length over all node combinations was referred to as the binary characteristic path length *L*^*b*^. The shortest path in the weighted network from node *i* to *j*was identified as the trajectory that minimized cost, with the “cost” of traveling a path taken as the inverse connection strength (Rubinov & Sporns, 2010). The weighted characteristic path length *L*^*w*^ was measured as the average shortest path cost between all node combinations. The weighted characteristic step length *Lstep*^*w*^ was defined as the minimal number of connections (i.e., discrete steps) the weighted shortest paths used and is a measure of the binary efficiency of weighted paths. Results for the weighted characteristic step length are presented in the Supporting Information. Both binary and weighted characteristic path lengths were compared with the characteristic path lengths *L*_*rand*_ and $L_{rand}^{w}$ of 10,000 randomized network versions, their ratio denoted by $L_{norm}^{b}$ and $L_{norm}^{w}$. To assess the contribution of the connection strength distribution on characteristic path length, the average characteristic path length $L_{shuffled}^{w}$ of 10,000 weights-shuffled network versions was examined.

*Modularity.* Modules in the binary and weighted networks were determined by modularity detection, generalized for directed and weighted networks (Leicht & Newman, 2008). The quality of the network division was expressed by the modularity *Q* (Newman, 2006). Similarity between the modularity structure of the binary and weighted network versions was quantified by the Rand index, measuring the probability of both community assignments being in agreement on grouping two regions together or apart (Rand, 1971). Statistical significance was assessed by computing the Rand index for 10,000 permutations with randomized modular assignments, and assigning a *p* value based on the proportion of permutations for which the Rand index exceeded the Rand index of the original dataset (Scholtens et al., 2014). Based on the modules in the binary network, connections were categorized as “intramodular” when spanning between nodes of the same module, and as “intermodular” when they connected regions belonging to different modules. Connectivity strength of both classes was compared and statistically evaluated by permutation testing by using random group assignment (10,000 permutations).

*Rich club organization.* Rich club organization of the network was assessed, exploring the tendency of high degree nodes to be more interconnected than expected on the basis of their individual degree alone (Colizza et al., 2006). With the Markov dataset providing information on in-degree of 91 cortical regions, rich club analysis was based on the number of afferent (incoming) axonal projection tracings from *all* 91 regions to the 29 regions of interest, referred to as *k*_*in*_. Binary rich club coefficient *ϕ*^*b*^ for *k_*in*_* was calculated as

$$\phi^{b}\left(k_{in}\right)=\frac{E_{>k_{in}}}{N_{>k_{in}}\left(N_{>k_{in}}-1\right)},$$

where $N_{>k_{in}}$ is the number of nodes in the 29 nodes network with an in-degree greater than *k*_*in*_, and $E_{>k_{in}}$ is the number of directed connections between these nodes. The weighted rich club coefficient *ϕ*^*w*^ (Opsahl et al., 2008) is the ratio of the total weight of connections between highest degree nodes and the sum of the strongest connections in the network, computed as

$$\phi^{w}\left(k_{in}\right)=\frac{W_{>k_{in}}}{\sum_{i=1}^{E_{>k_{in}}}w_{i}^{\text{rank}}},$$

where $W_{>k_{in}}$ is the total weight of connections between nodes with in-degree greater than *k*_*in*_, and *w*^*rank*^ are the ranked connection weights.

For both binary and weighted rich club coefficients, a normalized rich club coefficient *ϕ*_*norm*_ was derived by dividing rich club coefficients with the average rich club coefficient *ϕ*_*rand*_ of a set of randomized networks (10,000 randomized networks).

The probability that the normalized rich club coefficient *ϕ*_*norm*_(*k*) exceeded one due to chance was calculated as the percentage randomized networks with rich club coefficients exceeding the original coefficient *ϕ*(*k*). The resulting *p* values, assigned throughout the full range of *k*_*in*_, were corrected for multiple testing by the false discovery rate correction procedure (Benjamini & Hochberg, 1995).

The rich club was determined as the subset of the top 20% highest in-degree nodes (based on tracings from all 91 regions). Using the identified rich club, three connection classes were identified: connections spanning between rich club nodes (rich club connections), connections between rich club and peripheral nodes (feeder connections), and connections between peripheral nodes (local connections). Average strength of connection classes was compared in general, and among intra- and intermodular connections specifically (modules defined by the binary network). Statistical significance was assessed by means of permutation testing using random group assignment (10,000 permutations), false discovery rate correction was performed to adjust for multiple comparisons (Benjamini & Hochberg, 1995).

### Network Morphospace

In addition to comparing the observed clustering coefficient and characteristic path length with values seen in randomized networks, we compared them with minimum and maximum possible values for networks with the same density and connection strength distribution (Avena-Koenigsberger et al., 2014; Zamora-López & Brasselet, 2018). Specifically, we focused on the presence of large clustering and small characteristic path length that together indicates a small-world organization (Watts & Strogatz, 1998). We followed the procedures as proposed by Goñi et al. (2013) and Avena-Koenigsberger et al. (2014) to construct a “network morphospace” by using an evolutionary algorithm. Iteratively generated networks were optimized to two different types of network tradeoffs: (1) maximizing *L* and minimizing *C* to explore networks with weak (i.e., minimal) small-world organization and (2) minimizing *L* and maximizing *C* to explore networks with strong (i.e., maximal) small-world organization. Simulations were carried out on a population of 500 networks, initialized at the start with the original (binary and weighted both examined) macaque connectome. In each iteration, networks part of the so-called “Pareto front,” which means those networks that were most optimized, were identified as nondominated (following terminology introduced in Goñi et al., 2013), and networks not part of the Pareto front were identified as dominated. The networks in the population were optimized by iteratively replacing all dominated networks by (randomly selected) nondominated networks and in the replacement process four randomly selected network edges were adjusted. Networks in the morphospace of the binary network were constructed by randomly switching the four edges while preserving the in- and out-degree distributions (Rubinov & Sporns, 2010). We examined two types of morphospaces for the weighted network: in the first, the four network edges were randomly switched preserving the in- and out-degree and in-strength distributions (Rubinov & Sporns, 2010). In the second morphospace, connectivity strength of the four connections was randomly switched, preserving the binary topology and the connectivity strength distribution. The algorithm was applied for 1,000 iterations and the normalized characteristic path length and normalized clustering coefficient of the sampled networks provided a reference space to which the observed network was compared.

### Functional Dynamics

We further examined the role of connectivity strength in the macaque connectome by examining how the weights of the network shape functional dynamics. Brain network dynamics were simulated by the Kuramoto model with oscillators representing brain regions coupled according to structural pathways and functional dynamics estimated from the synchronization between oscillators (Cabral et al., 2014; Kuramoto, 1975; Rodrigues et al., 2016; Vuksanović & Hövel, 2014). In the Kuramoto model, the system evolves toward a globally synchronized system. With the macaque connectome involving a complex system of coupled values (i.e., binary and weighted topology), synchronization occurs in a nontrivial pattern that elucidates clusters of nodes that synchronize together and sets of nodes that drive the dynamical processes. The dynamics of the model based on the binary structural connectivity was defined by

$${\overset{\cdot }{\theta }}_{i}(t)=\omega_{i}+\lambda \overset{N}{\underset{j=1}{\sum }}A_{ji}\;\sin(\theta_{j}(t)-\theta_{i}(t))$$

where *θ*_*i*_(*t*) is the phase of the oscillator associated with brain region *i* at time *t*, and *ω*_*i*_ is the associated internal angular frequency and *N* is the number of brain regions. The cortical coupling strength is denoted by *λ* and indicates how much the phase of an oscillator is influenced by its neighbors. For the weighted structural network, coupling was described by the weighted connectivity matrix *W* normalized such that the total strength of all connections was equal to that of the binary connectivity matrix *A* to assure comparable coupling strength *λ* between networks (Motter et al., 2005). The model started with random initial phases, distributed uniformly in the interval [− *π*, *π*] and random internal frequencies, distributed uniformly between [0, 1]. In line with earlier studies, the evolution of the system was numerically approximated by the Runge-Kutta method for running time *T* = 700, and the system was evaluated after transient time *τ* = 300 (Schmidt et al., 2015). The model was evaluated for cortical coupling strengths in the range [0, 0.1] at intervals of 0.005. For the binary and weighted structural networks 1,000 model runs were realized for each cortical coupling strength, the functional dynamics of weights-shuffled network versions was obtained from model realizations of 1,000 weights-shuffled network versions.

The global dynamic coherence of the system was described by two order parameters *r* and *r*_*link*_ (Gómez-Gardeñes et al., 2007). The first order parameter *r* described the phase synchrony among all oscillators and was the time average of the modulus of the complex variable *z*(*t*) defined as,

$$z(t)=\frac{1}{N}\overset{N}{\underset{j=1}{\sum }}e^{i\theta_{j}(t)}.$$

The second order parameter *r*_*link*_ (Gómez-Gardeñes et al., 2007) was derived from the synchrony matrix *C*,

$$C_{ij}=\frac{1}{T-\tau }|\sum_{t=\tau }^{T}e^{i\left(\theta_{i}(t)-\theta_{j}(t)\right)}|,$$

and measures local construction of synchronization patterns by the fraction of synchronized node pairs

$$r_{link}=\frac{1}{N(N-1)}\underset{i,j}{\sum }C_{ij}.$$

The development of synchronization within the network was explored by examining the probability of region pairs being synchronized, defined as the average of the filtered synchrony matrix *F*,

$$F_{ij}=\left\{\begin{array}{ll} 1, & N\left(N-1\right)r_{link}\;\text{largest elements of}\;C\\ 0, & \text{lower values of}\;C \end{array}\right.,$$

over all model realizations of a specific cortical coupling strength.

The role of structural modules in synchronization was measured by the dynamical modularity, being the ratio between intra- and intermodular synchrony (Gómez-Gardeñes et al., 2010). Similarly, the effect of anatomical rich club structure on synchronization was examined by inspecting the ratio of synchrony among anatomical rich club regions and synchrony of other region pairs (including the synchrony between rich club regions and peripheral regions, and the synchronization among peripheral regions). The synchrony ratio of rich club regions and other region pairs was further investigated among intra- and intermodular region pairs separately.

## RESULTS

### Clustering

The binary macaque network exhibited significantly higher clustering (*C*^*b*^ = 0.76) than observed in randomized network instances ($C_{rand}^{b}=0.73\pm 0.0017$; *p* = 0.021), resulting in a normalized clustering coefficient of $C_{norm}^{b}=1.04$. Incorporating strength revealed a weighted clustering coefficient *C*^*w*^ of 3.70, significantly higher than observed in weighted randomized networks ($C_{rand}^{w}=3.42\pm 0.011$; *p* < 0.001), resulting in a normalized weighted clustering coefficient $C_{norm}^{w}=1.08$.

**Figure 2.**
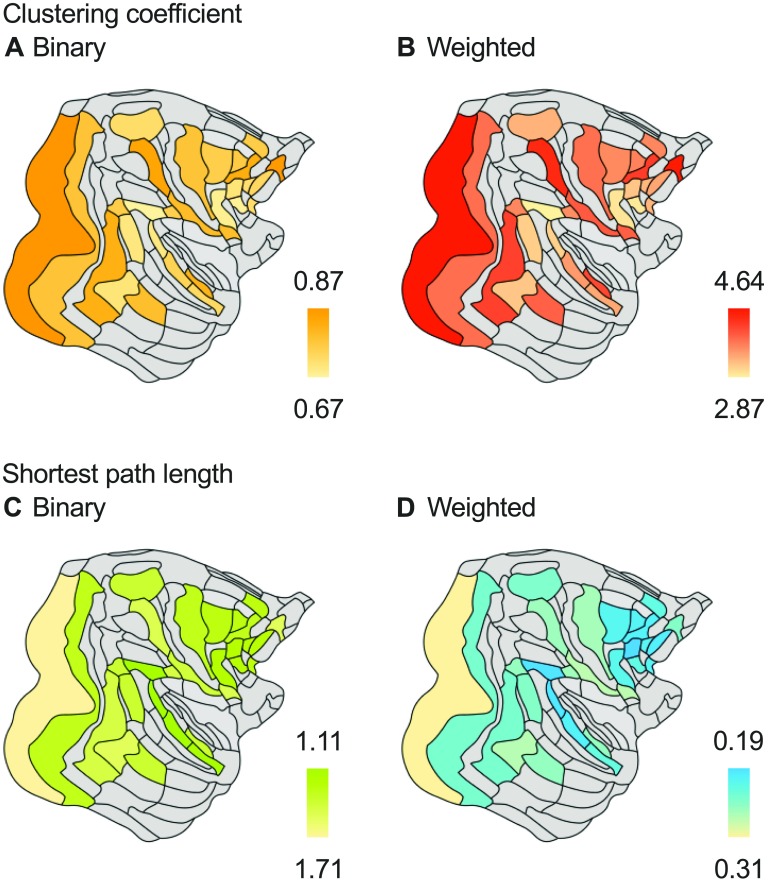
Clustering and characteristic path length per region. Clustering in binary (A) and weighted (B) macaque connectome reconstructions. Clustering coefficient of cortical regions in the binary network ranged from 0.67 (medium clustering) to 0.87 (high clustering). In the weighted reconstruction, clustering coefficients ranged from 0.54 (medium clustering) to 0.90 (high clustering). (C) Cortical regions showed characteristic path length ranging from 1.13 to 1.68 steps. (D) The path length of the weighted network ranged from 0.19 to 0.31 steps.

The weighted network showed significantly higher clustering coefficient (1.04×, *p* < 0.001) than network versions with projection strengths shuffled between connections ($C_{shuffled}^{w}=3.55\pm 0.011$). In the binary network, regions expressing high clustering (examining the top 20%) were distributed throughout the brain (Figure 2A), including regions in the occipital (V1, V4), prefrontal (8B, 10), parietal (5), and temporal cortex (PBr), and showed high overlap with the top 20% weighted clustering regions that included regions V1, V4, 8B, 10, 5, and region F7 (Figure 2B).

### Path Length

The characteristic path length of the binary network was short (*L*^*b*^ = 1.34) and not significantly different from the path length of the randomized networks ($L_{rand}^{b}=1.34\pm 0.00$; *p* = 0.86), resulting in a normalized path length of $L_{norm}^{b}=1.00$. The characteristic path length of the weighted macaque cerebral cortex network was *L*^*w*^ = 0.24 and was longer than for weighted randomized networks ($L_{rand}^{w}=0.21\pm 0.001$; *p* < 0.001), the normalized path length $L_{norm}^{w}$ being 1.16.

The connection strength distribution was found to increase the characteristic path length of the network, as network versions with shuffled projection strengths showed lower average path length than the original weighted network (0.87 ×, *p* = 0.001). Examining the average path length of regions revealed the top 20% regions exhibiting shortest path length to overlap between binary and weighted network (top regions in the binary network: frontal (F5), prefrontal (81, 9/46d, 8m), parietal (7A) and temporal cortex (STPc) (Figure 2C); top regions in the weighted network included areas in the prefrontal lobe (81, 9/46d, 9/46v, 8m), and two more posterior regions in the parietal (7A) and temporal lobe (STPc) (Figure 2D)).

### Network Morphospace

**Figure 3.**
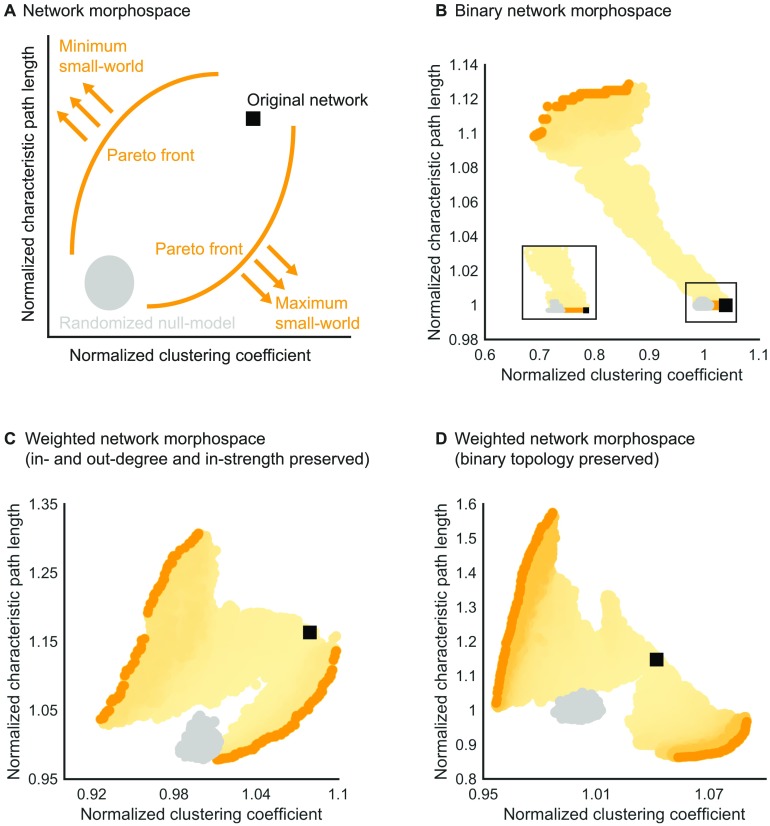
Network configurations in the small-world morphospace. (A) Schematic representation of a morphospace. The clustering coefficient and average shortest path length are shown for networks generated by either a small-world organization maximizing or minimizing optimization procedure (orange arrows). The black square indicates the original macaque connectome. Yellow circles indicate the generated networks, and darker shades of yellow indicate networks generated in later iterations. Network variants in the final iteration, forming the Pareto front, are indicated by orange circles. Gray circles represent the population of generated randomized networks. (B) The binary macaque network shows an as low as possible shortest path length and as high as possible clustering coefficient. (C) The weighted network shows close to the Pareto front of networks with maximum small-world organization in the morphospace of networks with similar in- and out-degree and in-strength. (D) The weighted network shows not close to either Pareto front in the space of networks with similar binary topology.

The clustering and path length of the macaque brain network was further examined in comparison with networks optimized for minimum or maximum small-world organization. All networks were graphically represented in a morphospace to elucidate the relative position of the macaque network in this network space (Figure 3). The morphospace of (degree-preserved) versions of the binary network showed normalized clustering coefficients with values between $C_{min}^{b}=0.69$ and $C_{max}^{b}=1.05$, and normalized characteristic path lengths between $L_{min}^{b}=1.00$ and $L_{max}^{b}=1.13$ (Figure 3B). The binary macaque network was located at the Pareto front of networks with optimized small-world organization and with a characteristic path length equal or lower than that of all generated networks.

The morphospace of (in- and out-degree and in-strength preserved) versions of the weighted macaque brain network showed normalized clustering coefficients with values between $C_{min}^{w}=0.90$ and $C_{max}^{w}=1.10$, and normalized characteristic path lengths between $L_{min}^{w}=0.95$ and $L_{max}^{w}=1.50$ (Figure 3C). The weighted macaque network was situated close to the Pareto front of networks with optimized small-world organization. Specifically, the macaque brain network was located on the right-side of the morphospace, indicating a relatively high clustering coefficient and relatively high average shortest path length. The proximity of the weighted network to the Pareto front illustrates that the high shortest path length (relative compared with randomized networks) was partly driven by mathematical constrains on networks with high clustering coefficients.

The morphospace of network versions with fixed binary topology and optimized connectivity strength distribution showed clustering coefficients that ranged from $C_{min}^{w}=0.99$ to $C_{max}^{w}=1.13$ and normalized path lengths between $L_{min}^{w}=0.87$ and $L_{max}^{w}=1.60$ (Figure 3D). The weighted macaque brain network was located in the middle of the Pareto fronts with minimum and maximum small-world organization, suggesting the connectivity strength distribution optimized small-world organization more than randomly distributed connectivity strength but that connectivity strength was not distributed to fully maximize, or minimize, the small-world organization.

### Modular Organization

**Figure 4.**
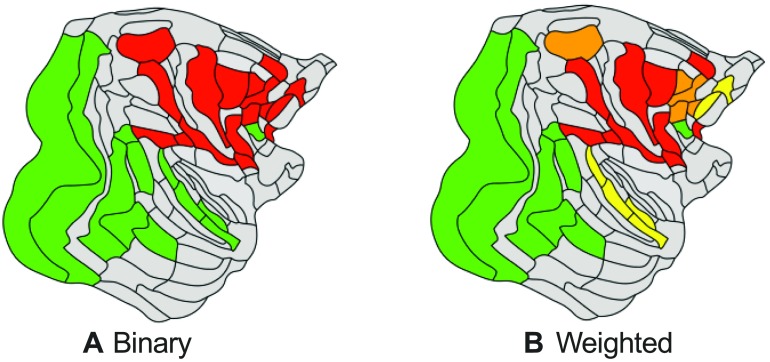
Detection of modules in the macaque dataset. (A) Modularity detection in the binary network showed the presence of two modules. (B) In the weighted network version, modularity detection revealed four modules. High overlap was observed between the modules found in the binary and weighted networks (Rand index = 0.70, *p* < 0.001, 10,000 permutations).

Modularity detection in the binary network (i.e., identifying modules based on connection density only) revealed two modules (*Q* = 0.10, Figure 4A). The largest module included regions in the prefrontal lobe (7 regions), frontal lobe (4), parietal lobe (6), and one region in the limbic lobe. The second module spanned all regions in the occipital lobe (4 regions), all regions of the temporal lobe (6), and one region in the prefrontal lobe. Thirty-eight percent of all connections were intermodular, linking different modules (50% of the possible intermodular node to node combinations), and 62% were intramodular (82% of the possible intramodular node to node combinations). Modularity detection in the weighted network (i.e., identifying modules based on connectivity strength) revealed a modularity structure consisting of four modules (*Q*_*w*_ = 0.21, Figure 4B). Two of the four modules overlapped with the modules identified in the binary network. Two new modules were distinct: the first module included mostly frontal/prefrontal regions (7m (parietal), F7 (frontal), 8B (prefrontal), 9/46d (prefrontal), 8m (prefrontal)) and the second included temporal/prefrontal regions (STPr (temporal), STPi (temporal), STPc (temporal), PBr (temporal), 10 (prefrontal), 46d (prefrontal)). Modular structure of the binary and weighted network showed high overlap (Rand index = 0.70, *p* < 0.001, 10,000 permutations). Examining connection strength with respect to the binary modules showed stronger intramodular connections than intermodular connections (1.40×, *p* < 0.001), suggesting connectivity strength to enhance the modular organization of the binary network.

### Rich Club Organization

**Figure 5.**
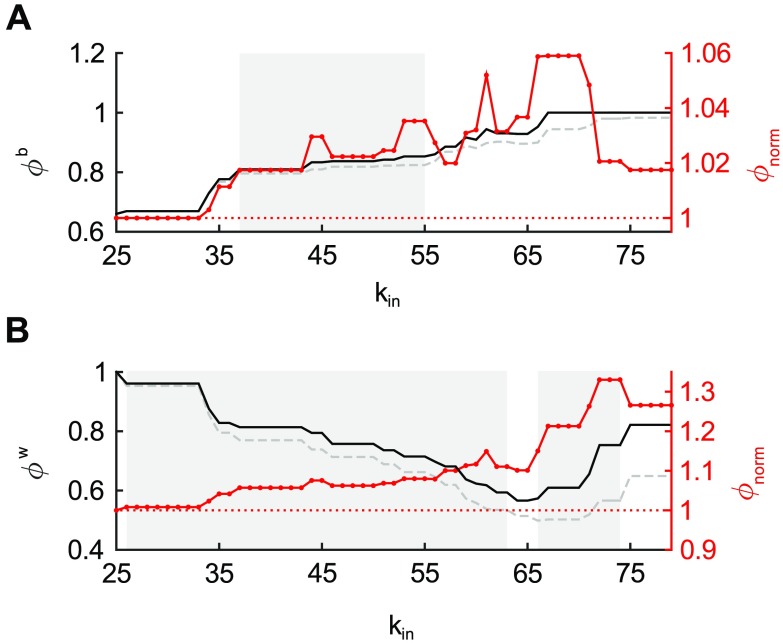
Rich club coefficient as function of in-degree *k*_*in*_. (A) Curves represent the rich club coefficient in the binary connectivity network of the macaque cerebral cortex (black), averaged rich club coefficient of randomized networks (gray; 10,000 permutations) and the normalized rich club coefficient (red). Shading of the region 36 <*k*_*in*_ < 56, indicates rich club presence where *ϕ*_*unw*_ significantly exceeded *ϕ*_*norm*_ (*p* < 0.05, FDR corrected). (B) For the weighted macaque network, the normalized rich club coefficient (red) increased with *k*_*in*_ and rich club organization was present for 25 <*k*_*in*_ < 64 and 65 <*k*_*in*_ < 75 (*p* < 0.05, FDR corrected).

The normalized binary rich club coefficient as a function of in-degree *k*_*in*_, computed on the incoming connections from all 91 regions, is shown in Figure 5. The rich club coefficient increased with *k_*in*_* and was significantly higher than random for 36 <*k*_*in*_ < 56 (*p* < 0.05, FDR corrected). Weighted rich club analysis revealed rich club organization throughout the range of in-degree *k*_*in*_ 25 <*k*_*in*_ < 64 and 65 <*k*_*in*_ < 75, *p* < 0.05 (FDR corrected, Figure 5).

Further examining rich club organization, we selected the top 20% highest in-degree nodes as hubs forming a rich club set (*k*_*in*_ ≥ 71) (Harriger et al., 2012). The selected six high in-degree regions included (ordered by in-degree) 81, 8m, 9/46d, 9/46v, F5, and 7m. Between these regions all possible binary connections were present, forming a fully connected clique. The weighted version of the rich club coefficient showed significantly higher connection strength between hub regions than expected from randomized networks (1.21×, *p* = 0.009), suggesting connection strength to underscore rich club organization. Moreover, the strength of connections linking rich club nodes was on average higher than strength of feeder (1.30×) and local connections (1.27×, *p* = 0.008 and *p* = 0.042, respectively, 10,000 permutations, all connection strength comparisons are FDR corrected). Strength of feeder and local connections were similar (*p* = 0.738).

**Figure 6.**
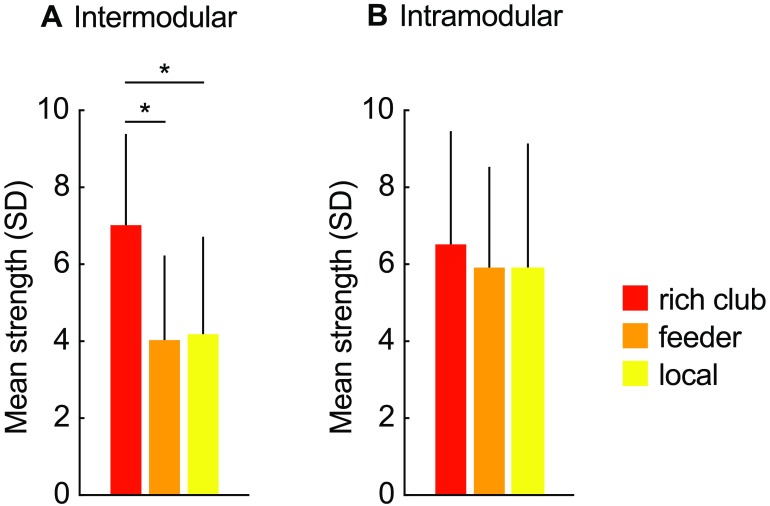
Comparison strength of connection groups. Average strength of inter- (A) and intramodular (B) connections, linking between rich club nodes (rich club connections, red), between rich club and the periphery (feeder connections, orange), or between peripheral regions (local connections, yellow). Among intermodular links, rich club connections exhibited on average strongest connections (rich club–feeder, *p* < 0.001; rich club–local, *p* < 0.004; feeder–local, *p* = 0.738; 10,000 permutations, FDR corrected), indicating the importance of rich club connections in communication between anatomical communities. Intramodular links showed similar strength between connection classes (all *p* > 0.05, FDR corrected).

Examination of rich club organization in modules showed that intermodular rich club connections were on average stronger than intermodular feeder connections present in the network (1.74×, *p* < 0.001, Figure 6) and intermodular local pathways (1.68×, *p* = 0.004, no significant difference was observed between strength of feeder and local connections, *p* = 0.738). Concerning the class of intramodular connections, rich club, feeder and local intramodular connections showed no significant strength differences (rich club–feeder: *p* = 0.607, rich club–local: *p* = 0.607, and feeder–local: *p* = 0.940).

### Functional Dynamics

The effect of the connectivity strength distribution on the modular and rich club organization observed in the macaque structural network was further investigated by examining simulated functional dynamics. The functional coherence of the networks was described by order parameters *r* and *r*_*link*_ and showed a critical period between *λ* = 0.02 and *λ* = 0.04 in which the functional networks switch from asynchronous dynamics to global synchrony.

**Figure 7.**
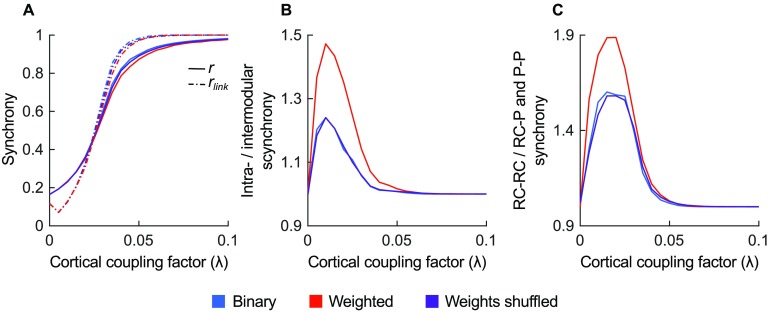
Synchrony of simulated function. (A) Computed average synchronization probability for binary, weighted, and weights-shuffled macaque networks showed a critical regime between 0.02 and 0.04. (B) Binary and weighted networks showed higher intramodular synchrony relative to intermodular synchrony, reflecting the structural organization. The intra-/intermodular synchrony ratio of the weighted network was also higher than in the weights-shuffled network, suggesting connectivity strength to increase local functional specialization. (C) The synchrony between rich club regions (RC-RC) was higher than between rich club regions and periphery (RC-P) or among peripheral regions (P-P) for the binary and weighted network. The synchrony ratio in the weighted network was higher compared with the ratio seen in the weights-shuffled network, indicating that the connectivity strength distribution increases network integration through rich club synchronization.

The synchronization between regions followed the structural modular organization, with higher synchronization observed within modules (based on the binary connectivity) than between modules for both dynamics simulated from binary and weighted structural networks (Figure 7). The ratio was particularly high for low cortical coupling values (maximum at *λ* = 0.01), suggesting synchronization within modules to precede intermodular synchronization. Comparing the functional dynamics of the weighted structural network and the weights-shuffled networks, showed higher intra- and intermodular synchrony ratio in the weighted network (0.005 ≤ *λ* ≤ 0.055, *p* < 0.001, FDR corrected), suggesting that connectivity strength in the macaque connectome endorses intramodular synchronization (Figure 7).

**Figure 8.**
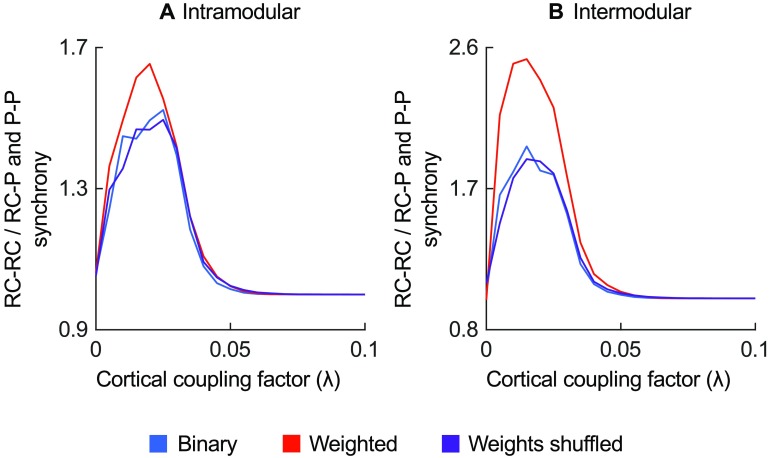
Interaction of rich club and modular network organization on synchrony of simulated function. The ratio of synchrony between rich club regions (RC-RC) and the synchrony between rich club regions and periphery (RC-P) or among periphery (P-P) among intramodular (A) or intermodular (B) region pairs.

In line with the rich club organization observed in the structural network, the simulated synchrony of both binary and weighted networks was higher among rich club regions than the synchrony between rich club regions and peripheral regions, and the synchrony among peripheral regions (Figure 7). Synchrony among rich club regions was higher in the weighted network than in weights-shuffled network versions (0.005 ≤ *λ* ≤ 0.045, *p* < 0.001, FDR corrected), suggesting the connectivity strength distribution also emphasizes functional rich club organization. Further inspection showed stronger rich club synchronization ratio between modules (maximum of 2.37) than within modules (maximum of 1.62, as shown in Figure 8).

## DISCUSSION

Our study provides extended evidence that the macaque connectome adheres to the general neural wiring principle of local specialization combined with systems-level topological integration. Graph analysis of the structural macaque connectome dataset obtained using tract-tracing by Markov et al. (2014) revealed that binary and weighted network versions have overlapping architectures, including a small-world, modular, and rich club organization. Investigation of the connectivity strength distribution showed projection strengths to enhance these network features in both structural and functional organization.

The macaque connectome showed architectural characteristics present in both binary and weighted connectivity networks. First, both networks showed local clustering and relatively short path lengths, together indicating a small-world network organization. Second, both networks showed a modular organization; two modules were reported in the binary network and four modules were revealed in the weighted network. Third, both network versions showed a rich club organization, with hub nodes (selected by in-degree) forming a densely connected rich club with a higher than chance mutual connectivity strength. These observations are in line with earlier examinations of the considered macaque connectome dataset (Bassett & Bullmore, 2017; Ercsey-Ravasz et al., 2013; Goulas et al., 2014; Markov et al., 2013a) and validate network analyses on earlier binary macaque connectome reconstructions (Bassett & Bullmore, 2006; Harriger et al., 2012; Hilgetag et al., 2000).

The strength placement across binary connections showed a nonrandom organization, with projection strengths increasing the binary network’s shortest path length and clustering (relative to randomized weight placement), with the observed network measures compared with the minimum and maximum these measures could attain. The combined increase in shortest path length, clustering, and modularity indicates a shift toward a stronger local network organization. Indeed, post hoc analysis confirmed that the characteristic step length increase resulted from stronger modular structure, with the weighted network showing lower characteristic step length within communities (1.46, *p* = 0.029) and higher step lengths between communities (1.90, *p* < 0.001) in comparison with weights-shuffled networks (respectively, 1.53 and 1.75). Rich club enhancement was significantly driven by effects in intermodular connections, suggesting that the distribution of connection strengths further boosts the rich club’s role as central infrastructure for global network integration (van den Heuvel & Sporns, 2013; Zamora-López et al., 2010).

Using functional simulations, we further showed that the connectivity strength distribution increases the modular and rich club organization. In the weighted network, intramodular synchronization preceded intermodular synchronization more strongly than in weights-shuffled reference networks, suggesting connectivity strength to be distributed toward local functional specialization (Sporns, 2011; Sporns et al., 2005). The rich club was more pronounced in leading global network synchronization in the weighted network than in weights-shuffled networks, suggesting connectivity strength to enhance the functional rich club as central integrator of neural information (Gómez-Gardeñes et al., 2010; Schmidt et al., 2015; Senden et al., 2014; Vlasov & Bifone, 2017). The role of the rich club in global functional integration was underscored by rich club regions leading intermodular synchronization more strongly than intramodular synchronization.

Our study provides evidence for a characteristic wiring organization in the macaque connectome, but the evolutionary, developmental, and neurophysiological principles that might underlie this organization remain unclear. Multiple, likely interacting, organizational principles have been proposed that determine the organization of neural networks (Vértes et al., 2012). Comparison of brain networks across species suggest a trade-off between topological integration and minimizing wiring length (van den Heuvel et al., 2016a; Song et al., 2014). Possible spatial constraints have also been observed in the macaque connectome in the form of an exponential relation between weights and distances (Ercsey-Ravasz et al., 2013). Furthermore, brain network architecture might also relate to repeated regional differentiation of brain regions during evolution, resulting in patterns of functional similarity and specialization of cortical areas (Ardesch et al., 2019; Betzel et al., 2015; Goulas et al., 2019; de Lange et al., 2016).

The following should be noted when interpreting the findings of the present study. First, the high density of the macaque connectome has a large impact on the topology of the binary macaque connectome, illustrated by small differences in the network measures between the observed macaque connectome and randomized network versions. Exploring the morphospace of the binary network revealed that as a result of the high density and degree distribution, the randomized versions of the network showed nearly minimum characteristic path length. This observation is in line with studies observing that the characteristic path length in randomized versions of cortical neural networks is not only short but even ultrashort, implying near-optimal efficiency of these randomized networks (Zamora-López & Brasselet, 2018). High network density also limits the possibly observable difference in normalized clustering coefficient between the binary network and randomized network versions ($C_{norm}^{b}=1.04$). The morphospace of the binary network showed that the clustering of the binary network was almost the maximum possible clustering ($C_{min}^{b}=0.69$ and $C_{max}^{b}=1.05$). This methodological limitation on the evaluation of the binary network topology has also been described and discussed earlier and argues for both more fine-grain parcellations and incorporating information on connectivity strength in network analyses (Bassett & Bullmore, 2017; Hilgetag & Goulas, 2015). The benefit of weighted connectome analysis was further underscored in the modularity analysis, in which incorporating connectivity strength in the module detection algorithm resulted in the detection of a finer modular structure (four modules) compared with the binary networks (two modules).

Second, analyses were constrained by the methodological limitations of the connectome reconstructions based on tract-tracing. The connectome map described full connectivity of 29 regions of 91 parcellated cortical regions, and future tract-tracing might complement the dataset. Specifically, the reconstructed connectivity described intrahemispheric connections, and the inclusion of interhemispheric connections could provide more understanding of the whole-brain network organization. Variation in tracer uptake across injections might affect the number of labeled neurons reported. We validated our results to be independent of tracer uptake by repeating our analyses with connectivity strength weighted by fraction of extrinsic labeled neurons (FLNe). The FLNe weighting provides a relative measure of connectivity strength that normalizes the total tracer uptake across injections. The FLNe connection strength showed strong correlation with the number of labeled neurons strength (*ρ* = 0.99, *p* < 0.001, Spearman’s rank correlation of raw nonzero values), and we observed in all our analyses similar results when using the FLNe measure, underlining the robustness of our results (see Supporting Information).

Third, the investigated Kuramoto model for functional dynamics models all brain regions as identical oscillators. This is a simplification, with studies showing neurophysiological heterogeneity in the cortex having clear influences on functional connectivity (Chaudhuri et al., 2015; van den Heuvel et al., 2016b; Mejias et al., 2016; Turk et al., 2016). Moreover, in the used Kuramoto model the system eventually evolves to a globally synchronized state. Future studies could incorporate global time delays to examine the effect of structural connectivity strength on meta-stable functional connectivity. Combined research into structural connectivity, functional connectivity, and neuroarchitecture might provide further understanding of function and organization of large scale neural networks (Wang & Kennedy, 2016).

We observe that strength of connections in the macaque connectome is nonrandomly distributed and enhances local processing and global network integration. Our findings elucidate the role of connectivity strength in the topology of the macaque connectome.

## ACKNOWLEDGMENTS

We thank Fraukje Coopmans for her input on earlier versions of the manuscript.

## Supporting Information

Supporting information for this article is available at https://www.doi.org/10.1162/netn_a_00101.

## AUTHOR CONTRIBUTIONS

Siemon C. de Lange: Investigation; Methodology; Writing - Original Draft; Writing - Review & Editing. Dirk Jan Ardesch: Methodology; Writing - Original Draft; Writing - Review & Editing. Martijn P. van den Heuvel: Conceptualization; Funding acquisition; Investigation; Methodology; Project administration; Supervision; Writing - Original Draft; Writing - Review & Editing.

## FUNDING INFORMATION

Martijn P. van den Heuvel, MQ: Transforming Mental Health (GB). Martijn P. van den Heuvel, Nederlandse Organisatie voor Wetenschappelijk Onderzoek (NL), Award ID: VIDI-452-16-015. Martijn P. van den Heuvel, Nederlandse Organisatie voor Wetenschappelijk Onderzoek (NL), Award ID: ALWOP.179.

## TECHNICAL TERMS

Connectome: The comprehensive network of neural interactions of a species nervous system.
Modular organization: Property of network nodes to form communities with strong within- community connectivity but relatively weak connectivity to nodes of other communities.
Hubs: Set of brain regions with high number of connections.
Rich club: Set of hub regions that are more densely interconnected than expected based on their degree alone.
Small-world organization: The presence of both local and global integration in a network.
Kuramoto model: Mathematical model describing synchronizations between coupled oscillators as model for the functional dynamics between brain regions.
Clustering coefficient: Ratio between existing and possible number of triangle motifs in a network. Measure of local network circuitry.
Characteristic path length: Average minimum number of steps (or minimal weight) needed to travel between any two nodes in a network. Measure of global network integration.
Network morphospace: Representation of networks in a space with axes that represent specific network properties.
Tract-tracing: Method to reconstruct axonal projections by using viral tracers.
