# Supplementary material for: Connection strength of the macaque connectome augments topological and functional network attributes
Source: Netw Neurosci. 2019 Sep 1;3(4):1051–69. doi: 10.1162/netn_a_00101 (PMC6777983; doi:10.1162/netn_a_00101)

de Lange, S. C., Ardesch, D. J., van den Heuvel, M. P. (2019). Supporting information for "Connection strength of the macaque connectome augments topological and functional network attributes." *Network Neuroscience*, *3*(4), 1051–1069. https://doi.org/10.1162/netn_a_00101

Supplementary Material for the article

Connection strength of the macaque connectome in relation to topological network attributes

Siemon C. de Lange[1], Dirk Jan Ardesch[1] and Martijn P. van den Heuvel[1,2*]

[1] *Connectome Lab, Department of Complex Trait Genetics, Center for Neurogenomics and Cognitive Research, Vrije Universiteit Amsterdam, Amsterdam Neuroscience, Amsterdam, The Netherlands*

[2] *Department of Clinical Genetics, Amsterdam UMC, Vrije Universiteit Amsterdam, Amsterdam Neuroscience, Amsterdam, The Netherlands*

Path length

The characteristic step length of the weighted macaque cerebral cortex network was $Lstep^w$ = 1.71 and was longer than for weighted randomized networks ($Lstep^w_{rand} = 1.66 \pm 0.01$; $p < 0.001$), the normalized path length $Lstep^w_{norm}$ being 1.03. The connection strength distribution was found to increase the characteristic step length of the network, as network versions with shuffled projection strengths showed slightly lower average step length than the original weighted network (0.98×, $p = 0.001$).

FLNe weighted network analysis

The FLNe weighted network reported a clustering coefficient above levels seen in randomized networks (1.62× higher, $C^w = 0.0052$ and $C^w_{rand} = 0.0032 \pm 8.74\times10^{-5}$; $p < 0.001$). The shortest path length of the FLNe weighted network was computed using resistance, $r_{ij} = -\ln(w_{ij})$, as connection cost function. The characteristic path length showed higher than levels seen in randomized networks (1.33×, $L^w = 7.00$ and $L^w_{rand} = 5.26 \pm 0.10$; $p < 0.001$).

The normalized clustering coefficient and normalized characteristic path length of the FLNe weighted network were further examined with respect to the morphospace of network versions with preserved in- and out-degree and in-strength, and in the morphospace of network versions with preserved binary topology and connectivity strength distribution (Figure SI 1). The network morphospaces showed very similar and in both morphospaces the FLNe weighted network was located on the Pareto front of networks with maximal small-world organization. Specifically, the FLNe weighted network was located on the upper part of the Pareto front, indicating that the FLNe weighted network is a network with very high clustering coefficient that, mathematically, cannot have a low shortest path length (i.e. path length equal to values observed in randomized null-models).

The modularity detection algorithm distinguished 5 modules with modularity measure Q = 0.63. With respect to the binary modules, the FLNe weighted modules showed stronger intramodular connections than intermodular connections (5.81×, $p < 0.001$).

The weighted rich club coefficient did not show significantly higher in the FLNe network compared to the randomized network versions. Connections were distinguished into three classes –rich club, feeder and local connections– based on the binary topology and feeder connections. Intermodular rich club connections were stronger than feeder connections (23.1×, $p = 0.010$) and stronger than local connections (1.23×, $p = 0.010$), but no differences were observed between intermodular local and feeder connections ($p = 0.54$). For intramodular connections no significant differences were observed between rich club and feeder connections ($p = 0.72$) or rich club and local connections ($p = 0.72$), but local connections showed stronger than feeder connections (2.24×, p = 0.009).

Simulated functional synchronization based on FLNe weighted connection strength, displayed a critical period between 0 and 0.2 in which the order parameters r and r_{link} showed a network transition from asynchronous dynamics to global synchrony. Synchronization was higher intramodular than intermodular (maximum ratio of 2.75) and the effect was stronger

than seen in weights shuffled reference networks. The maximum ratio occurred at $\lambda = 0.03$ at the start of the critical period suggesting intramodular synchronization precedes global synchronization. The synchronization between rich club regions was higher than between rich club and peripheral regions or between peripheral regions (maximum ratio of 1.76, Figure SI 2) and higher than seen in weights shuffled reference networks. The maximum ratio was observed at the start of the critical period ($\lambda = 0.03$), suggesting a leading role for the rich club in global network synchronization. Interaction showed stronger rich club synchronization ratio intermodular (maximum 4.71) than intramodular (maximum 1.68, Figure SI 3).

Figure SI 1. Network configurations in the small-world morphospace. The normalized clustering coefficient and normalized average path length of the FLNe weighted network is examined with respect to the morphospace of networks with similar in- and out-degree and in strength (a) and of networks with similar binary topology and connectivity strength distributions (b).

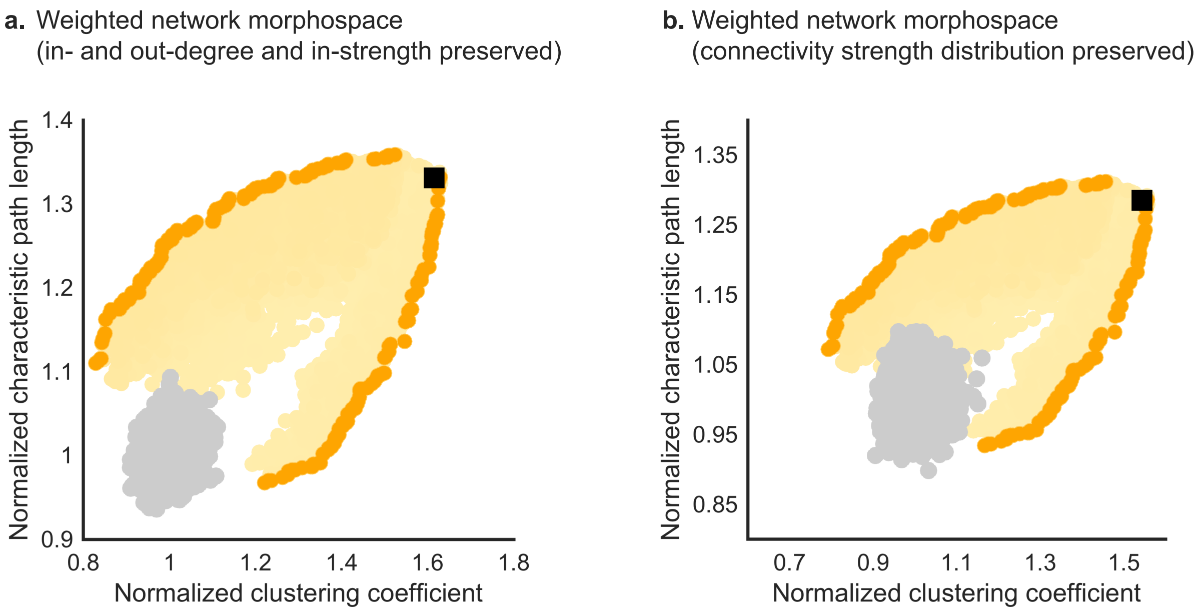

Figure SI 2. Synchrony of simulated function (a) Computed order parameters r and r_{link} of the binary, weighted and weights-shuffled macaque networks showed a critical regime of the network dynamics between 0.02 and 0.04. (b) The FLNe weighted network showed higher intramodular synchrony compared with intermodular synchrony than observed in weights-shuffled network versions. (c) The ratio between synchrony among rich club regions (RC – RC), and the synchrony between rich club regions and periphery (RC – P) or among periphery (P – P) showed higher in the FLNe weighted network compared with weights-shuffled network versions.

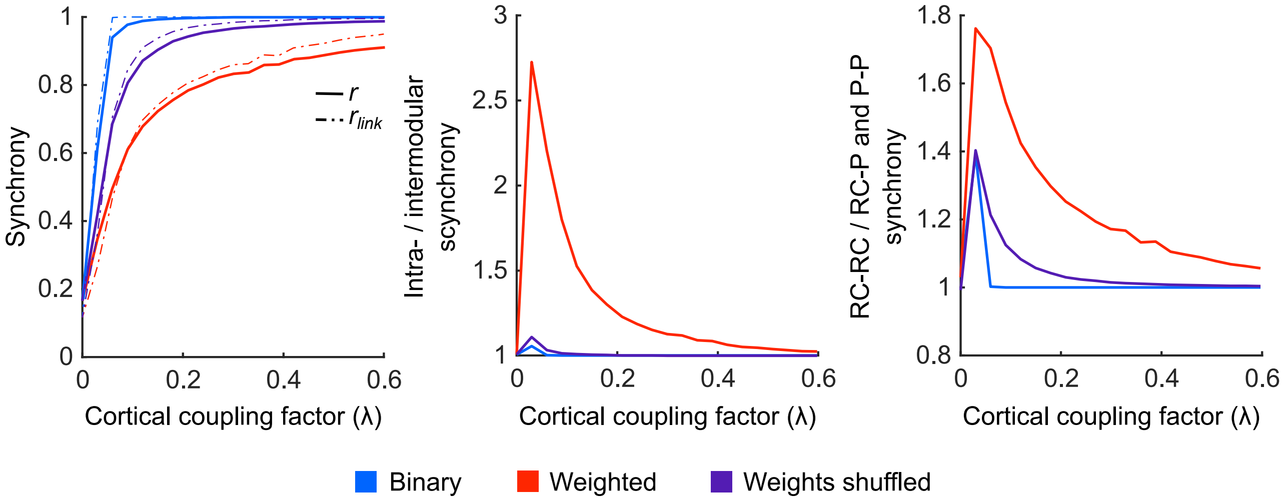

Figure SI 3. Interaction of rich club and modular network organization on synchrony of simulated function. The ratio of synchrony between rich club regions (RC – RC) and between rich club and periphery (RC – P) or among periphery (P – P) for the weighted network and weights-shuffled network. The ratio showed stronger intermodular (a) than intramodular (b).

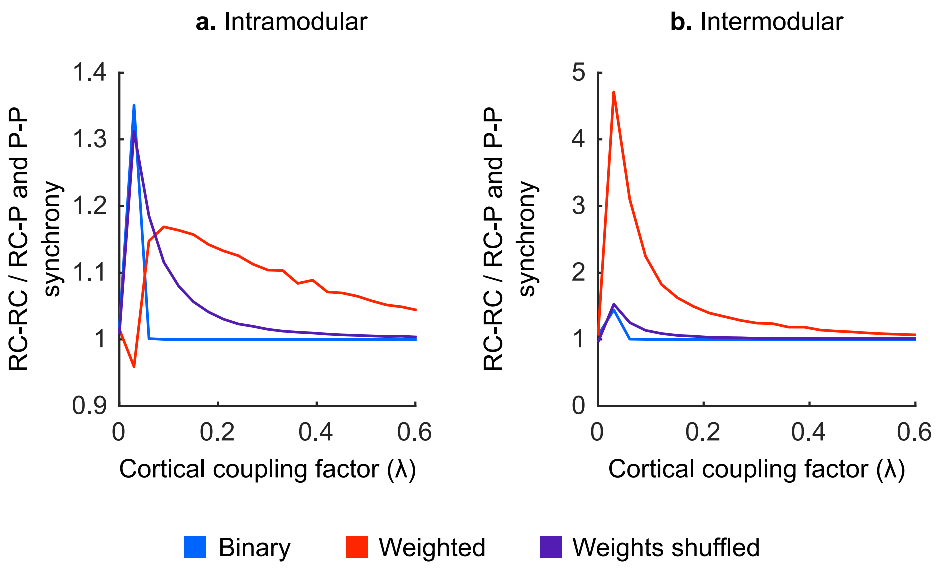

Supplement: Supplementary file 1 [file netn-03-1051-s001.pdf]
